# Vague data analysis using neutrosophic Jarque–Bera test

**DOI:** 10.1371/journal.pone.0260689

**Published:** 2021-12-02

**Authors:** Muhammad Aslam, Rehan Ahmad Khan Sherwani, Muhammad Saleem

**Affiliations:** 1 Department of Statistics, Faculty of Science, King Abdulaziz University, Jeddah, Saudi Arabia; 2 College of Statistical and Actuarial Sciences, University of the Punjab Lahore, Lahore, Pakistan; 3 Department of Industrial Engineering, Faculty of Engineering-Rabigh, King Abdulaziz University, Jeddah, Saudi Arabia; University of Defence in Belgrade, SERBIA

## Abstract

In decision-making problems, the researchers’ application of parametric tests is the first choice due to their wide applicability, reliability, and validity. The common parametric tests require the validation of the normality assumption even for large sample sizes in some cases. Jarque-Bera test is among one of the methods available in the literature used to serve the purpose. One of the Jarque-Bera test restrictions is the computational limitations available only for the data in exact form. The operational procedure of the test is helpless for the interval-valued data. The interval-valued data generally occurs in situations under fuzzy logic or indeterminate state of the outcome variable and is often called neutrosophic form. The present research modifies the existing statistic of the Jarque-Bera test for the interval-valued data. The modified design and operational procedure of the newly proposed Jarque-Bera test will be useful to assess the normality of a data set under the neutrosophic environment. The proposed neutrosophic Jarque-Bera test is applied and compared with its existing form with the help of a numerical example of real gold mines data generated under the fuzzy environment. The study’s findings suggested that the proposed test is effective, informative, and suitable to be applied in indeterminacy compared to the existing Jarque–Bera test.

## 1 Introduction

The standard statistical tests from the parametric domain play a vital role in decision-making problems and are popular in social sciences [1]. The outcomes of the tests are considered reliable and valid for the population under investigation. These parametric tests help understand the research problems for better decision-making, prediction, and estimation purposes. The fruits of the tests are only juicy when the standard assumptions under the parametric tests validate. One of the standard assumptions of these tests is the validation of the normality assumption. The analysis and recommendations without checking the normality of the data mislead the decision-makers. Several tests have been proposed to assess the normality of data. Jarque-Bera (JB) test is famous goodness of fit test used to assess the distributional structure of data. The validation of the normality assumption under the JB test relies on the principle of matched skewness and kurtosis of the sample data with the normal distribution. The test is applied for testing the null hypothesis that there is no significant difference between the data in hand and the normal distribution versus the alternative hypothesis that a significant difference exists. Several authors applied this test in various fields. [2] discussed the power of the JB test. [3] presented the modification of the JB test. [4] discuss the power of various statistical normality tests. [5] worked on the modification of the JB test for the multivariate data. [5] applied the JB test for the face recognition problem. More details about the test and other analyses can be seen in [2, 6–11].

One of the JB test restrictions is the computational limitation available only for the data in exact form. When the observations in the data are fuzzy, the existing JB test under classical statistics cannot be applied for testing the normality of the data. The data based on the fuzzy logic are often in the interval-valued form [12, 13]. An extension of the fuzzy logic and interval-based approach is called the neutrosophic logic [14]. The neutrosophic logic can provide information about the measure of indeterminacy. [15] introduced the neutrosophic logic. The other forms of fuzzy sets are singular valued fuzzy sets [16], hesitant fuzzy sets [17], Zadeh fuzzy sets [18], intuitionistic fuzzy sets [19] etc. The applications of the neutrosophic logic can be seen in [20–36]. Based on neutrosophic logic, neutrosophic statistics were introduced by [37]. Neutrosophic statistics is the generalization of classical statistics applied when the data is measured from a complex or indeterminate environment. References [38, 39] provided the methods to analyze the data having neutrosophic numbers. Reference [40] introduced the area of neutrosophic statistical quality control. References [41, 42] introduced tests of normality under neutrosophic statistics. The details about the neutrosophic statistics can be seen in [43, 44]. [45] applied the JB test using the fuzzy approach in forecasting solar radiation. Reference [46] applied this test for prediction stock closing prices. [47] preened a novel distance measure method and applied it in gold mines data. For more details, the reader may read [48–60].

Motivating from the computational limitations of the existing JB test for the exact form data, we proposed a modified version of the present JB test for the fuzzy or interval-valued data. The proposed JB test is a generalized form of the existing JB test from classical statistics as it possesses the ability to deal with both exact and fuzzy forms data sets. Gold mines data is one of the data sets like water level, temperature, stock exchange, melting points, etc., that may possess the indeterminate or neutrosophic form. We will present the application of the proposed test with the help of gold mines data taken from [47]. The efficiency of the proposed JB test will be compared with the existing test. The use of the developed JB test will be beneficial in situations where the observations under a problem are not certain, fuzzy, indeterminate, interval-valued, or in neutrosophic form.

## 2 Preliminary

Let z_N_ = a_N_+b_N_I_N_; I_N_ϵ[I_L_, I_U_] be a neutrosophic random variable with determinate part a_N_ and indeterminate part b_N_I_N_. Note here that the neutrosophic random variable becomes the traditional random variable if I_L_ = 0. Suppose that n_N_ϵ[n_L_, n_U_] be neutrosophic sample size. By following [38], the neutrosophic average for z_N_ϵ[z_L_, z_U_] is given as

$${\bar{z}}_{N}={\bar{a}}_{N}+{\bar{b}}_{N}I_{N};\;I_{N}\epsilon [I_{L},\;I_{U}]$$ (1)

where ${\bar{a}}_{N}=\frac{1}{n_{N}}\sum_{i=1}^{n_{N}}a_{i}$ and ${\bar{b}}_{N}=\frac{1}{n_{N}}\sum_{i=1}^{n_{N}}b_{i}$

The neutrosophic difference between z_N_ and ${\bar{z}}_{N}$ is given as

$$(z_{N}-{\bar{z}}_{N})=(a_{N}-{\bar{a}}_{N})+(b_{N}-{\bar{b}}_{N})I_{N}$$ (2)

The neutrosophic sum of square (NSS) is given by

$$\sum_{i=1}^{n_{N}}{\left(z_{i}-{\bar{z}}_{iN}\right)}^{2}=\left[\begin{array}{c} \min\left(\begin{array}{c} \left(a_{i}+b_{i}I_{L})(\bar{a}+\bar{b}I_{L}),\;(a_{i}+b_{i}I_{L})(\bar{a}+\bar{b}I_{U}\right)\\ \left(a_{i}+b_{i}I^{U})(\bar{a}+\bar{b}I_{L}),\;(a_{i}+b_{i}I_{U})(\bar{a}+\bar{b}I_{U}\right) \end{array}\right)\\ \max\left(\begin{array}{c} \left(a_{i}+b_{i}I_{L})(\bar{a}+\bar{b}I_{L}),\;(a_{i}+b_{i}I_{L})(\bar{a}+\bar{b}I_{U}\right)\\ \left(a_{i}+b_{i}I_{U})(\bar{a}+\bar{b}I_{L}),\;(a_{i}+b_{i}I_{U})(\bar{a}+\bar{b}I_{U}\right) \end{array}\right) \end{array}\right],\;I\epsilon [I_{L},I_{U}]$$ (3)

The neutrosophic measure of skewness k_3N_ϵ[k_3L_, k_3U_] is given as

$$k_{3N}=\frac{\sum_{i=1}^{n_{N}}{\left(z_{i}-{\bar{z}}_{iN}\right)}^{3}}{nS_{N}^{3}};\;S_{N}\epsilon [S_{L},\;S_{U}]$$ (4)

The neutrosophic measure of kurtosis k_4N_ϵ[k_4L_, k_4U_] is given as

$$k_{4N}=\frac{\sum_{i=1}^{n_{N}}{\left(z_{i}-{\bar{z}}_{iN}\right)}^{4}}{nS_{N}^{4}};\;S_{N}\epsilon [S_{L},\;S_{U}]$$ (5)

Note here that S_N_ϵ[S_L_, S_U_] presents the neutrosophic standard deviation and defined as follows

$$S_{N}=\sqrt{\sum_{i=1}^{n_{N}}{\left(z_{i}-{\bar{z}}_{iN}\right)}^{2}/n_{N}};\;S_{N}\epsilon [S_{L},\;S_{U}],\;n_{N}\epsilon [n_{L},\;n_{U}]$$ (6)

## 3 Jarque–Bera test under neutrosophic statistics

The JB test is used to confirm the normality of a data set before applying the famous standard statistical tests like t-test, z-test, or F-test. The test is based on the null hypothesis there is no difference between the data under study and the normal distribution versus the alternative hypothesis that difference exists. The test statistic of the JB test is a function of skewness and kurtosis. Suppose S, K and n are the sample skewness, kurtosis and the sample size for a data set then the statistic used for JB test under classical statistics is defined as:

$$JB=\frac{n}{6}\left[S^{2}+\frac{1}{4}{\left(K-3\right)}^{2}\right]$$ (7)

The test-statistic will be useful when the data are in exact form and helpless in the case of interval-valued data. We modify the JB-statistic defined in (7) for the interval-valued data. Now, the modified JB test under neutrosophic environment used the null hypothesis H_0N_ that the neutrosophic data is the same as the neutrosophic normal distribution versus the alternative hypothesis H_1N_ that the neutrosophic data is different from the neutrosophic normal distribution. The operational procedure of the proposed JB test under neutrosophic statistics is stated in the following steps:

**Step-1:** Select a neutrosophic random sample of size n_N_ϵ[n_L_, n_U_]. Compute the averages of the determined part a_i_(i = 1,2,…,n_L_) and indeterminate part b_i_(i = 1,2,…,n_U_) as follows

$${\bar{a}}_{N}=\frac{1}{n_{N}}\sum_{i=1}^{n_{N}}a_{i}\;\mathrm{and}\;{\bar{b}}_{N}=\frac{1}{n_{N}}\sum_{i=1}^{n_{N}}b_{i}$$ (8)

**Step-2:** The neutrosophic average of a neutrosophic random variable is calculated as

$${\bar{z}}_{N}={\bar{a}}_{N}+{\bar{b}}_{N}I_{N};\;I_{N}\epsilon [I_{L},\;I_{U}]$$ (9)

**Step-3:** The difference between z_N_ and ${\bar{z}}_{N}$ will be computed as

$$(a_{N}-{\bar{a}}_{N})+(b_{N}-{\bar{b}}_{N})I_{N}$$ (10)

**Step-4:** Compute the sum of square (SS) as follows

$$\sum_{i=1}^{n_{N}}{\left(z_{i}-{\bar{z}}_{iN}\right)}^{2}=\left[\begin{array}{c} \min\left({\left(a_{11}-{\bar{a}}_{1}\right)}^{2},\;((a_{11}-{\bar{a}}_{1})(\left(a_{11}-{\bar{a}}_{1})+1\times (b_{11}-{\bar{b}}_{1}\right)),\;(a_{11}-{\bar{a}}_{1})+1\times {\left(b_{11}-{\bar{b}}_{1}\right)}^{2})\right)\\ \max\left({\left(a_{11}-{\bar{a}}_{1}\right)}^{2},\;((a_{11}-{\bar{a}}_{1})(\left(a_{11}-{\bar{a}}_{1})+1\times (b_{11}-{\bar{b}}_{1}\right)),\;(a_{11}-{\bar{a}}_{1})+1\times {\left(b_{11}-{\bar{b}}_{1}\right)}^{2})\right) \end{array}\right]$$ (11)

**Step-5:** Compute k_3N_ϵ[k_3L_, k_3U_] and k_4N_ϵ[k_4L_, k_4U_].
**Step-6:** Compute JB statistic under neutrosophic statistic JB_N_ϵ[JB_L_, JB_U_] using the following formula

$$JB_{N}=n_{N}\left(\frac{{\left(k_{3N}\right)}^{2}}{6}+\frac{{\left(k_{4N}\right)}^{2}}{24}\right);\;JB_{N}\epsilon [JB_{L},\;JB_{U}],\;k_{3N}\epsilon [k_{3L},\;k_{3U}],\;k_{4N}\epsilon [k_{4L},\;k_{4U}]$$ (12)

The statistic JB_N_ proposed in (12) follows asymptotically to a Chi-square distribution with two degrees of freedom. The normal distribution has a skewness zero and kurtosis three indicates that for a normal distribution, the value of JB_N_ is zero and any excess value of JB_N_ from zero will indicate the deviation from normality.

**Step-7:** choose the tabulated value from the Chi-square table and accept H_0N_ if JB_N_ϵ[JB_L_, JB_U_] less than the tabulated value at the level of significance α.

## 4 Application in cleaner production data

This section will present the computational aspects of the proposed methodology of the newly developed JB test under a neutrosophic environment. The application of the proposed JB_N_ test is given with the help of cleaner production data from the gold mines. [47] discussed the cleaner production data for gold mines based on experts’ evaluation evidence under fuzzy theory. The availability of the gold mines data under fuzzy logic motivates us to use the data for the application purposes for the present research. According to [47], the decision-maker is interested in selecting a suitable center from the three centers C_1_, C_2_ and C_3_ on the basis of five characteristics of gold mines data. [47] presented a comprehensive way to select the best center based on their decision criteria but did not perform the normality test before using the methods. To test the data normality, we will use only the characteristic management level C_1_. According to [47], "it indicates the production process and equipment level, which contains the mining technology and production equipment. The data of gold mines of the characteristics G_1_, G_2_ and G_3_ of center C_1_ is selected from [47] and reported in Table 1. It can be seen that the data is in the indeterminacy interval; therefore, we will apply the proposed test to check the normality of the data first.

**Table 1:** The gold mines data of characteristic C_1_.

G_1_		G_2_		G_3_	
a_i_	b_i_	a_i_	b_i_	a_i_	b_i_
0.32	0.43	0.1	0.18	0.22	0.4
0.09	0.15	0.13	0.23	0.12	0.1
0.22	0.31	0.29	0.32	0.28	0.39
0.13	0.44	0.08	0.5	0.23	0.4

The proposed test using the real data for three centers G_1_, G_2_ and G_3_, respectively is implemented as follows

**Step-1:** Select a neutrosophic random sample of size n_N_ϵ[4,4]. The averages of the determined part a_i_(i = 1,2,…,n_L_) and indeterminate part b_i_(i = 1,2,…,n_U_) are:

${\bar{a}}_{N}=\frac{1}{4}\sum_{i=1}^{4}a_{i}=0.19$ and ${\bar{b}}_{N}=\frac{1}{4}\sum_{i=1}^{4}b_{i}=0.3325$

${\bar{a}}_{N}=\frac{1}{4}\sum_{i=1}^{4}a_{i}=0.15$ and ${\bar{b}}_{N}=\frac{1}{4}\sum_{i=1}^{4}b_{i}=0.3075$

${\bar{a}}_{N}=\frac{1}{4}\sum_{i=1}^{4}a_{i}=0.2125$ and ${\bar{b}}_{N}=\frac{1}{4}\sum_{i=1}^{4}b_{i}=0.3225$

The statistics indicate the average performance of the three methods G_1_, G_2_ and G_3_ laid down by the experts with respective average indeterminacy levels for the selection of the gold mines center, e.g., the average performance of the cleaner production gold mines for the G_1_ method is 0.19 with a 0.3325 average uncertainty level.

**Step-2:** The neutrosophic averages of neutrosophic random variables are given as

${\bar{z}}_{N}=0.19+0.3325I_{N}$; I_N_ϵ[0,0.05]

${\bar{z}}_{N}=0.15+0.3075I_{N}$; I_N_ϵ[0,0.05]

${\bar{z}}_{N}=0.2125+0.3225I_{N}$; I_N_ϵ[0,0.05]

**Step-3:** The difference between z_N_ and ${\bar{z}}_{N}$ for example for G_1_ is given by

$z_{N}-{\bar{z}}_{N}=[0.13,0.1348],\ldots ,[-0.06,-0.0546]$

**Step-4:** Compute the sum of square (SS) for three centers are as follows

$\sum_{i=1}^{n_{N}}{\left(z_{N}-{\bar{z}}_{N}\right)}^{2}=[0.0314,0.1338]$, [0.0274,0.0792], [0.0134,0.1337]

**Step-5:** The neutrosophic values of k_3N_ϵ[k_3L_, k_3U_] and k_4N_ϵ[k_4L_, k_4U_] for three centers are given as

k_3N_ϵ<[0.3623, −0.0755], [1.000,0.0323], [−0.6113, −0.3565]>

k_4N_ϵ<[−1.3797, −2.5762], [−0.7911, −2.6052], [−0.9277, −2.7399]>

**Step-6:** The calculated values of statistic JB_N_ϵ[JB_L_, JB_U_] are given as

JB_N_<[0.4047,1.1099], [0.7711,1.1319], [0.3926,1.3359]>

The value of the proposed JB_N_ test statistic is not much far away from zero, indicating that the gold mines data follow a normal probability distribution with an indeterminacy level. The same can be verified by using the Chi-square distribution table in Step-7.

**Step-7:** The table value for the level of significance 0.05 is 7.815. We note that JB_N_ϵ[JB_L_, JB_U_] are less than the tabulated value. Therefore, the null hypothesis is that data from three centers are not significantly different from the neutrosophic normal distribution, and this decision is the same as [47].

## 5 Comparative study

In this section, the performance of the proposed test will be compared with the JB test under classical statistics. The proposed JB_N_ϵ[JB_L_, JB_U_] defined in Eq (12) is the extension of the existing JB test presented in Eq (7). The proposed test will be reduced to the JB test under classical statistic if JB_N_ = JB_L_ = 0. The neutrosophic form of the proposed JB_N_ϵ[JB_L_, JB_U_] test for centers G_1_, G_2_ and G_3_ along with the measures of indeterminacy are shown in Table 2.

**Table 2:** The measures of indeterminacy associated with the test.

Centers	JB_N_ϵ[JB_L_, JB_U_]	Measure of Indeterminacy	Neutrosophic form	Existing JB test
G_1_	[0.4047,1.1099]	I_N_ϵ[0,0.6353]	JB_N_ = 0.4047+1.1099I_N_	0.4047
G_2_	[0.7711,1.1319]	I_N_ϵ[0,0.3187]	JB_N_ = 0.7711+1.1319I_N_	0.7711
G_3_	[0.3926,1.3359]	I_N_ϵ[0,0.7061]	JB_N_ = 0.3926+1.3359I_N_	0.3926

From Table 2, we note that the measure of indeterminacy is increased if the gap between JB_N_ϵ[JB_L_, JB_U_] is increased. We also note that the proposed test provides the measures of indeterminacy, while the existing JB test under classical statistics cannot provide this kind of information. For example, when the level of significance is 5%, according to the proposed JB test under neutrosophic statistics, the probability that the null hypothesis is accepted is 0.95, and the null hypothesis is rejected with the probability of 0.05. Other than these probabilities, the chance that the decision-makers are uncertain about the acceptance or rejection of the null hypothesis is 0.6353. We note that the sum of the probabilities is larger for the proposed test, and this theory is the same as in [37]. The proposed test can be compared with the existing JB in terms of sensitivity. From Table 2, it can be seen that values of the classical test (determined part) fluctuate much as compared to the indeterminate part. For example, for centers G_2_ and G_3_, the values of the existing JB test moves from 0.4047 to 0.7711 when measure of indeterminacy changes from 0.6353 to 0.3187. On other hand, the values of the indeterminate part of the proposed JB test moves from 1.1099*I*_*N*_ to 1.1319*I*_*N*_ when measure of indeterminacy changes from 0.6353 to 0.3187. From the study, it can be seen that the proposed test is less sensitive than the existing JB test. We also note that the proposed test provides the results in indeterminacy intervals and makes it suitable and effective to be applied in the indeterminate environment. This theory is the same as in [38, 39].

## 6 Concluding remarks

The paper extends the concept of the Jarque–Bera test from classical statistics to neutrosophic statistics. The classical JB test is limited to perform on exact values data. In contrast, the proposed modified form of the JB statistic can be used to both exact and interval-valued data. The design and operational procedure for the newly developed JB test are presented under the fuzzy and neutrosophic logic. The application of the proposed JB test is carried on the real data set from the cleaner production of gold mines generated in a fuzzy environment. Moreover, a comparison of the proposed neutrosophic JB test is made with the existing JB test to assess the performance of the two tests. The findings of the numerical example suggested that the proposed JB test is effective, informative, and suitable to be applied under indeterminacy compared to the existing JB test. For generalized and better analysis of the data, the proposed test is recommended when the data is obtained from indeterminate and complex systems. The proposed methodology of the JB test can be extended to test the multivariate normality under indeterminacy. The proposed test for big data can be extended for future research. The development of new software to perform the proposed test is a fruitful area for future research.
